# Supplementary material for: Bacillus subtilis PS-216 Antagonistic Activities against Campylobacter jejuni NCTC 11168 Are Modulated by Temperature, Oxygen, and Growth Medium
Source: Microorganisms. 2022 Jan 26;10(2):289. doi: 10.3390/microorganisms10020289 (PMC8875091; doi:10.3390/microorganisms10020289)
Supplement: Supplementary file 1 [file microorganisms-10-00289-s001.zip › microorganisms-1540593-supplementary.pdf]

Supplementary information

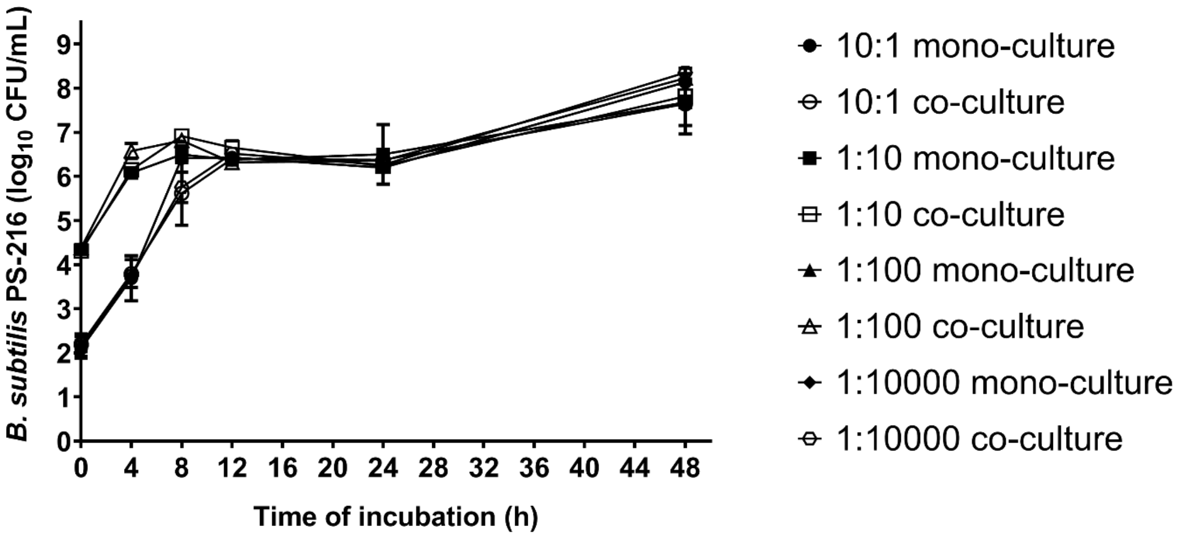

Figure S1. Growth of *B. subtilis* PS-216 in mono-culture and in co-culture with *C. jejuni* 11168, in 10:1 in favor of *B. subtilis,* and 1:10, 1:100 and 1:10000 in favor of *C. jejuni* in the starting inoculum in MH broth at 42°C in microaerophilic conditions, presented as log_{10}CFU/mL ± standard deviation of three replicas.

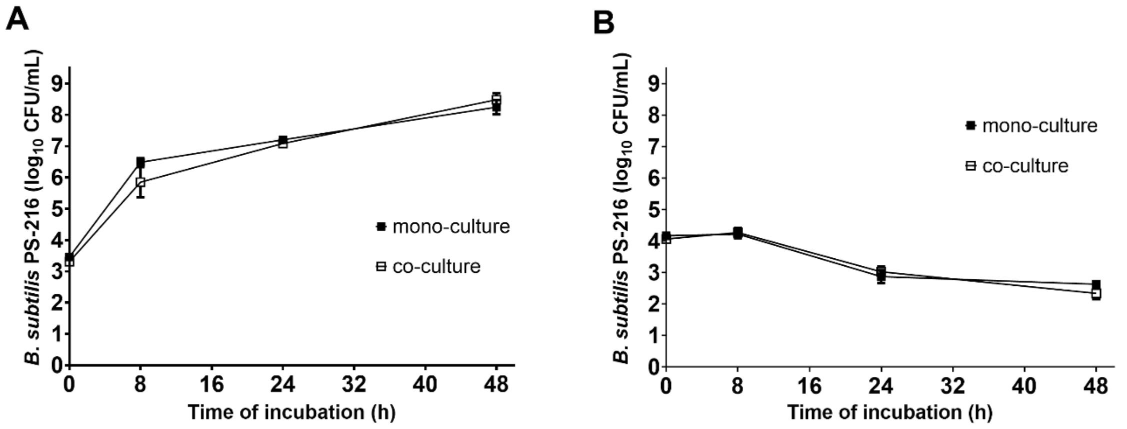

Figure S2. Growth of *B. subtilis* PS-216 in sterile chicken litter medium (A) and sterile intestinal content medium (B), in mono-culture (full symbols) and co-culture (empty symbols), presented as log_{10}CFU/mL ± standard deviation of three replicas.
